# Public consultation in the evaluation of animal research protocols

**DOI:** 10.1371/journal.pone.0260114

**Published:** 2021-12-01

**Authors:** Michael W. Brunt, Daniel M. Weary

**Affiliations:** Animal Welfare Program, Faculty of Food and Land Systems, University of British Columbia, Vancouver, Canada; Chang-Qing Gao, Central South University, Xiang Ya Hospital, CHINA

## Abstract

One response to calls for increased openness in animal research is to make protocols publicly accessible, but it is unclear what type of input the public would provide if given this opportunity. In this study we invited public responses to five different research projects, using non-technical summaries intended for lay audiences. Our aim was to assess the potential for this type of public consultation in protocol review, and a secondary aim was to better understand what types of animal research people are willing to accept and why. US participants (*n* = 1521) were asked (via an online survey) “Do you support the use of these (insert species) for this research”, and responded using a seven-point scale (1 = “No”, 4 = “Neutral”, and 7 = “Yes”). Participants were asked to explain the reasons for their choice; open-ended text responses were subjected to thematic analysis. Most participants (89.7%) provided clear comments, showing the potential of an online forum to elicit feedback. Four themes were prevalent in participant reasoning regarding their support for the proposed research: 1) impact on animals, 2) impact on humans, 3) scientific merit, and 4) availability of alternatives. Participant support for the proposed research varied but on average was close to neutral (mean ± SD: 4.5 ± 2.19) suggesting some ambivalence to this animal use. The protocol describing Parkinson’s research (on monkeys) was least supported (3.9 ± 2.17) and the transplant research (on pigs) was most supported (4.9 ± 2.02). These results indicate that public participants are sensitive to specifics of a protocol. We conclude that an online forum can provide meaningful public input on proposed animal research, offering research institutions the opportunity for improved transparency and the chance to reduce the risk that they engage in studies that are out of step with community values.

## Introduction

Public views towards the use of animals in scientific research are multidimensional [1] and can be influenced by purpose [2], species [3] and procedure [4]. At least one public representative is required on committees that evaluate animal-based research protocols in many oversight systems including Canada [5] and the United States [6, 7]. Given the diverse public views surrounding animal research, it seems unlikely that one person can effectively represent the “public”. Within the current regulatory framework, lay committee members of Animal Care and Use Committees can feel outnumbered and intimidated by institutional and scientific members [8].

Animal Care and Use Committees attempt to address animal welfare concerns through the 3Rs; replacement of animals with non-animal models, reduction of the number of animals used in experiments and refinement of procedures to minimize pain and distress [9]. However, this focus on the 3Rs can supplant a broader discussion on societal values [10] that may help institutions retain social license [11] for the research. Public consultation has the potential to ensure that societal norms are better reflected in research, but it is not clear what approaches are likely to be effective. In previous studies, we have experimented with using online tools to garner public opinion about different types of animal research [4, 12], but the very process of asking participants their views requires explaining what research is proposed and the reasons why it is done. This led us to consider the ways in which animal protocols are currently evaluated and approved, and whether there might be opportunity to increase the transparency of animal research through public consultation as part of protocol review.

Our objective was to assess the potential of providing non-technical summaries for public consultation and to better understand what types of animal research people are willing to accept and why.

## Methods and materials

This project was approved by the University of British Columbia’s Behavioural Research Ethics Board (H20-01863).

### Experimental design

Our survey (S1 File) was accessed by participants through Qualtrics, a cloud-based survey platform. Each participant was randomly allocated to one of five animal research protocols, each based on published research, varying in level of invasiveness, the type of animals used, and the purpose of the study. The five hypothetical studies were 1) chronic pain research using mice, 2) transplantation research using pigs, 3) smoking research using mice, 4) Parkinson’s research using monkeys, and 5) skin cancer research using zebrafish. Participants were asked, “Do you support the use of these (insert species) for this research”. They were asked to indicate their response using a seven-point scale, with 1 indicating “No”, 4 indicating “Neutral”, and 7 indicating “Yes”. Participants were then asked to describe the reasons for their response by typing text in a text box. The survey ended with a series of general questions on attitudes towards animals.

The survey was first tested for clarity with 16 test participants from the University of British Columbia. All responses provided by these participants were then removed before launching the experiment. A fit-to-census-data sample of paid U.S. participants was recruited from July 31 to August 3, 2020 using the participant-sourcing platform CloudResearch (Table 1). Of the original 2874 completed surveys, 1323 failed an attention check (directed to select “other” and type: I read instructions) and 30 completed the survey in less than 142 seconds (half the medium duration to complete the survey) leaving 1521 completed surveys.

**Table 1:** Participant demographic targets vs actual recruitment.

Demographic	Category	Census Target	Actual	Difference from Census (%)
**Total Participants**		1521	1521	
**Gender**	Woman	775	835	7.74
	Other	746	686	-8.04
**Age (years)**	18–34	455	479	5.27
	35–54	500	491	-1.80
	55+	566	551	-2.65
**Income**	Less than $35,000	433	420	-3.00
	$35,000 to $74,999	453	502	10.82
	$75,000 to $149,999	420	418	0.48
	$150,000 and above	215	181	-15.81
**Region**	Midwest	317	302	-4.73
	South	581	582	0.17
	Northeast	261	292	11.88
	West	362	345	-4.70

### Analysis

Qualitative data were analyzed by qualitative description [13]. A pilot study formed the bases of *a priori* themes [14], in which codes emerged through constant comparison and axial coding [15]. The author (MB) coded 247 participant responses across all five research scenarios (NVivo, version 12.3.0, QSR International Pty Ltd.) and the codebook formed as parent codes were organized into emergent themes of 1) impact on animals, 2) impact on humans, 3) scientific merit, and 4) availability of alternatives. These themes were used as *a priori* themes in the current study. Inter-coder reliability and codebook validity was established by a one author (MB) and another researcher who independently coded a subset of 200 responses [following 16]. Substantial agreement was reached between the two researchers (Kappa = 0.79) and consensus was reached on all remaining coded differences. The initial researcher (MB) then coded all remaining participant responses with the final codebook (S2 File). Illustrative quotations were selected based on how effectively these related to the theme. Participants were assigned anonymous numbers upon entry to the survey which are associated with the quotes in the text. Quotations that required editing for clarity are indicated using square brackets around inserted words.

Quantitative data were analyzed using linear regression (GLM procedure in SAS, version 9.04, SAS Institute Inc.). The effects of protocol, participant demographic factors including gender (woman v. other), age (18–34 v. 35–54 v. 55 and older), income (under $35,000 v. $35,000–74,999 v. $75,000–149,999 v. $150,000 and above), parent (yes v. no), and pet owner (yes v. no), and the interaction between scenario and each of the demographic factors were considered in the single model. The normality of residuals was visually assessed.

## Results

### Qualitative responses

On average participants provided 17 words (minimum of 1 word and maximum of 139 words) to justify their response to the animal research protocols. 10.3% of participants did not offer sufficient detail (for example: “I’m neutral” or “It’s ok”); all other participants offered justifications that provided sufficient detail to classify comments into the *a priori* themes. The four themes regarding support for the research were: 1) impact on animals, 2) impact on humans, 3) scientific merit, and 4) availability of alternatives (Table 2).

**Table 2:** Themes present in participant (*n* = 1521) reasoning regarding their support for five different research protocols, together with the number of participants (expressed both as a whole number and as a percentage of the sample) who expressed this theme.

Themes^1^	no.^2^	%
**Impact on animals**	727	47.8
**Impact on humans**	339	22.3
**Scientific merit**	207	13.6
**Availability of alternatives**	181	11.9

^1^ 156 participant responses did not offer sufficient detail to categorize to theme.

^2^ Some responses contained more than one theme, so total number of themes referenced was greater than the number of participants.

#### Impact on animals

Participant responses centered on both individual animals and populations. Responses focused on the experience of individuals often referenced pain, suggesting that it was immoral to hurt others. For example, participant 363 stated: *“It seems like you are purposely causing pain to a living creature and it does not seem ethical”*. Reasons focused more on populations of animals sometimes referenced a harm-benefit evaluation (e.g. that the study would do more harm than good or vice versa). Some responses sought to downplay harms (or benefits) as part of their larger calculus. In the words of one participant: *“Although I support treating animals humanely*, *this small number of pigs doesn’t compare to the mass produced for [agricultural] slaughter*. *I support science advancements and trust these trials to improve overall wellbeing*.*”* (3317). Some responses also sought to downplay the value of the animals’ lives. For example, participants wrote: *“They are disgusting rodents”* (1113), *“They’re just fish and don’t have emotions like other animals”* (3042), *“I don’t like monkeys”* (3350), and *“Animals are to serve humans”* (1042).

#### Impact on humans

Some responses described the impact on humans, including benefits to humans such as curing disease, drug development, improving treatments, human safety or saving lives. For example, one participant wrote *“This is important research which would benefit mankind*. *Many good outcomes would hopefully follow from this work [including] saving lives and improving health…”* (568). Some participants referred to the detrimental impact of the disease on a person, family or society (e.g. *“Parkinson’s is rather debilitating and the drugs used to treat it have awful side effects*. *The only way we can learn more about the disease and how the medications affect the patients is by doing research*.*”* 906). Personal experiences were also cited in some responses. One participant wrote: *“I know people with skin cancer and if this can help save lives I am 100% for it”* (129).

#### Scientific merit

The perceived value and quality of the proposed research was noted by participants, including the perceived relevance (e.g. for scientific advancement) and the appropriateness of the animals used. Some participants described the research as unnecessary (e.g. *“It’s pretty clear the adverse effects smoking has on pregnancy and children…It just seems like an unnecessary study…”* 253), but others stressed the importance of scientific understanding (e.g. *“Any advancement of science and knowledge is valuable to human life”* 1948). Respondents sometimes described the animal species as a good model (e.g. *“Mice share similar DNA to humans and studies using mice [have] helped humans make many scientific advancements…”* 1286), but others questioned this (e.g. “…*I am concerned that the results of any drug trial would not translate properly from a species [fish] as far removed from humans…*” 3279).

#### Availability of alternatives

The concept of alternatives arose in four forms: 1) some participants suggested that there were no suitable alternatives to the proposed research (e.g. *“Unfortunately*, *for some research there is no other way to find pertinent information except for animal testing”* 3103); 2) others suggested that suitable alternatives were available (e.g. *“…Some people have these problems and it’s just silly to try and replicate problems in mice when you could be helping people as you learn more”* 1342); 3) or that alternatives should be investigated (e.g. *“I’m not an animal activist but there must be a better way to find a drug than killing monkeys”* 951); 4) or that the research could be avoided by pursuing social policy alternatives; in the smoking scenario, some respondents justified low levels of support by suggesting that society instead seek to limit the use of tobacco (e.g. *“I will not be willing to support this research because I feel like smoking should be taken away all together…”* 2853).

### Quantitative analysis

Quantitative responses for the proposed research ranged between 1 and 7, with a mean ± SE of 4.5 ± 0.06 indicating ambivalence towards the proposed research. Despite the variation, some proposals were more supported than others (F_4, 1504_ = 9.19, *p*<0.0001). Participants were most supportive of the transplantation research using pigs (4.9 ± 0.12) and least supportive of the Parkinson’s disease research on monkeys (3.9 ± 0.12), with intermediate responses to the proposed studies on skin cancer research using zebra fish (4.7 ± 0.12), chronic pain research using mice (4.6 ± 0.13), and the effects of smoking using mice (4.4 ± 0.13).

**Fig 1:**
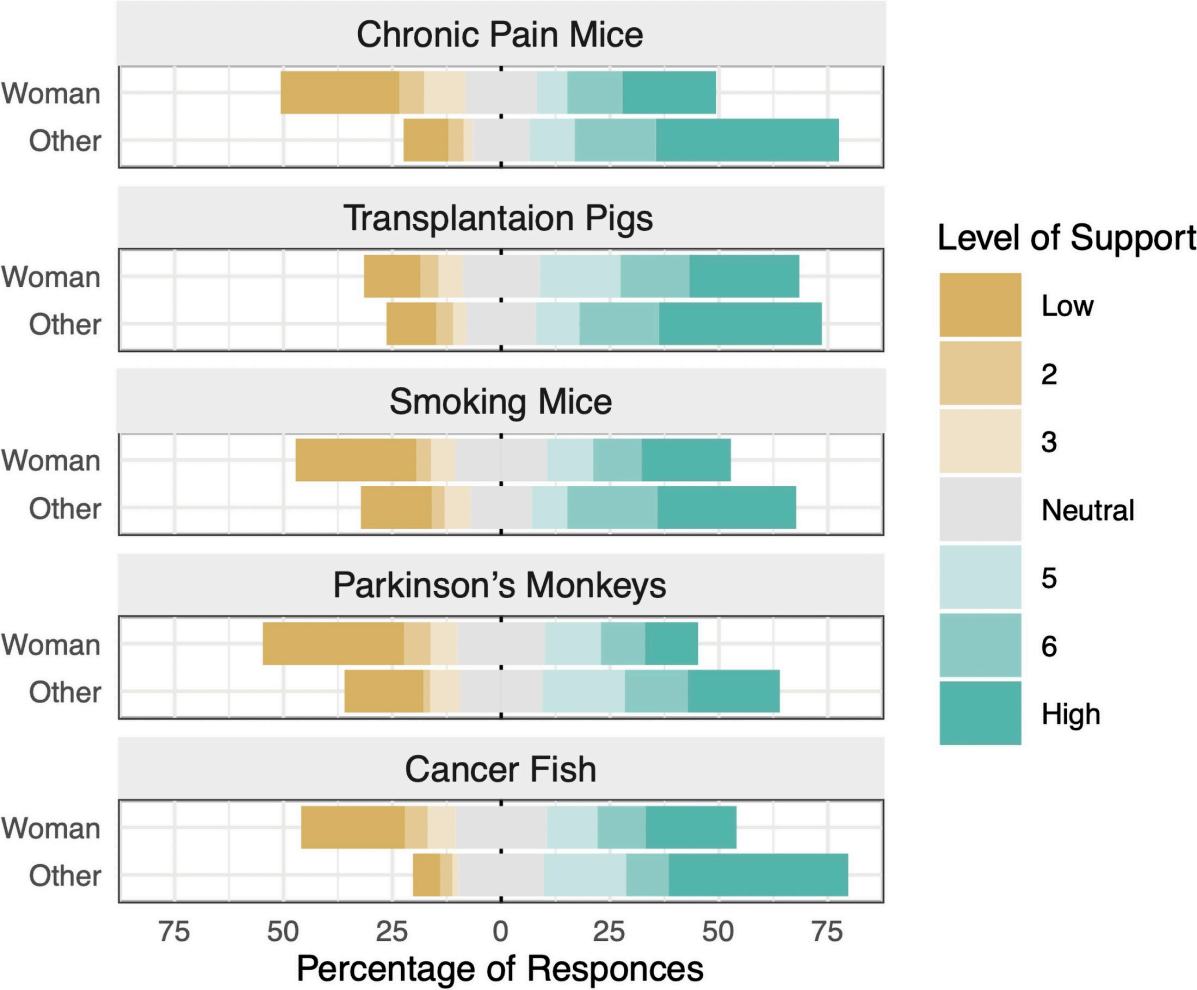
Distribution of participant (*n* = 1521) support (1 = “No” to 7 = “Yes”) for research proposals describing chronic pain research with mice, organ transplant research with pigs, smoking research with mice, Parkinson’s disease research with monkeys, and cancer research with zebrafish. Results are shown separately for participants who identified as female versus those with any other gender identity.

Five demographic variables explained some of the variation in support for the proposed research protocols (Table 3); participants identifying as women (F_1, 1504_ = 60.44, *p*<0.0001), who were younger (F_2, 1504_ = 8.11, *p* = 0.0003), with lower incomes (F_3, 1504_ = 8.02, *p*<0.0001), without children (F_1, 1504_ = 7.54, *p* = 0.0061), but with pets (F_1, 1504_ = 5.63, *p* = 0.0177) were less supportive. There was an interaction between the effect research proposal and gender, with women showing low levels of support to all scenarios except the transplant study on pigs and those not identifying as women showing higher levels of support for all but the Parkinson’s research using monkeys (F_4, 1504_ = 2.45, *p* = 0.0442; Fig 1).

**Table 3:** Participant (n = 1521) score by demographic questions known to influence the support of animal research after responding to one of the five research proposals.

Demographics	Response options	Likert score (mean ± SE)
**Gender**	Woman	4.0 ± 0.08
	Other	5.0 ± 0.08
**Age**	19–34	3.9 ± 0.10
	34–54	4.7 ± 0.10
	54+	4.8 ± 0.09
**Income**	Less than $35,000	4.0 ± 0.11
	$35,000 to $74,999	4.4 ± 0.10
	$75,000 to $149,999	4.8 ± 0.10
	Above $150,000	5.1 ± 0.15
**Parent**	Yes	4.7 ± 0.08
	No	4.2 ± 0.08
**Pets**	Yes	4.4 ± 0.07
	No	4.7 ± 0.10

## Discussion

There have been calls for increased transparency in animal research, both from the public and the scientific community [17]. Giving more information about rules and regulations tends to improve public attitudes towards animal research [18, 19], and while increasing transparency is generally welcomed, to date broad societal input has not been included during the evaluation of proposed research involving animals. The public is the primary funder (via taxes) and consumer of research outcomes (e.g. via new medicines) so some societal input is warranted [17], especially given the weakness associated with current procedures intended to elicit public viewpoints [20]. The current study shows that, when provided the opportunity to comment on animal research proposals, the majority of participants (~90%) provided comments with sufficient detail to explain their stance. Institutions that conduct studies on animals (and perhaps other forms of ethically contentious research) could integrate this information into institutional decision-making processes. This input could be used to identify problematic areas of research (e.g. those causing severe suffering) and thus reduce the risk that research practices fall out of step with community values.

The culture within Animal Care and Use Committees is influenced by its members; as many committees are composed mostly of scientists, priority is often placed upon scientific interpretations [21]. Community or public members are typically outnumbered by scientific members on these committees [22], and these members can feel limited in their ability to provide input [8]. The online consultation method modelled in the current study captured a wide diversity of public input that could aid in the evaluation of research protocols beyond the current scope of the 3Rs. For example, many comments discussed the moral permissibility of this form of animal use, an important issue for committees to consider.

Participants were sensitive to the specifics of each protocol. In general, scenarios involving chronic pain in mice, organ transplantation in pigs and skin cancer in fish all exhibited higher levels of support than what is typically reported for animal research [23, 24]. Our qualitative analysis suggests that the perceived scientific value and benefit to humans were reasons participants tended to support these proposals. Conversely, opposition to the proposed studies was often rooted in questions concerning the generalizability and translatability of the research. There is growing literature documenting how well (or poorly) animal-based research translates into new therapeutic treatments for humans [25–29]. The findings of the current study suggest that public support for research will be influenced by information on the likelihood that the research results in the intended benefits.

In response to the research proposals involving mice, pigs, and fish (and less so for monkeys) some participants expressed a lack of concern for the experience of the animal, sometimes related to perceived differences in the psychological and physical characteristics of the species concerned [30]. Previous studies have shown that people sometimes rank species along a socio-zoological [31] or societal sentience scale [32]. Our findings are consistent with broader research showing that attitudes vary with species [33], and suggest that species will influence protocol evaluation.

One reason for opposition to the animal research was a belief that alternative approaches should be adopted. The belief that non-animal alternatives exist has been shown to reduce participant support for animal use [3, 34]. Conversely, support is known to increase if participants perceive there is no alternatives to animal use [3, 35]. Describing the methods used to evaluate alternatives could help the public in deciding whether to support or reject the proposed research.

Opposition to the smoking study using mice was largely a result of participants viewing the research as unnecessary. This result is consistent with previous work reporting that research seen to be unnecessary has little support [36]. Participant responses also illustrate how the concept of ‘alternatives’ can include more than just non-animal models such as in-vitro tests; this concept extends to different ways of addressing the social issue (e.g. arguing that resources would be better spent helping people quit smoking). Thus public consultation on animal use should include information about the availability of social policy alternatives.

Women were less likely to support the research proposals. A number of earlier studies have shown that women are less likely to support the use of animals in research [34, 37], consistent with a variety of studies that report gender differences in attitudes towards animals [35, 38–40]. The gender by research proposal interaction detected in the current study also suggests that women consider certain types of harm especially egregious, or are more cautious in their assessment of the benefits; additional studies are encouraged to determine the basis for these gender differences. This result also emphasizes the importance of considering gender composition in animal care and use committees.

If institutions conducting research on animals choose to consult the public during protocol review, our online study suggests that feedback from participants can provide nuanced and thoughtful explanations for their support or lack thereof. Current research governance practices typically offer few opportunities to incorporate societal concerns [10]. Furthermore, the current governance structures often fail to recognize that public concerns can be reasonable and worthy of serious consideration [41].

The current study does not address the question of what type of interaction and involvement are desired (or considered sufficient) by the public. Fitting a multidimensional public into one definitive public opinion narrative is not easily accomplished [42]. However, these challenges should not dissuade institutions from public consultation. Our model provided participants an effective means to express their support and concerns.

The current study has several limitations. We employed a census-based representative sample of US participants, limiting our ability to generalize to other geographical regions. We specifically encourage new research on participants from other geographical areas such as Canada and the UK, that also require public participation in protocol review [10, 43]. Institutions using animals in research may be especially interested in attitudes within their local community. For example, a university might wish to consult views from within their state, city, university, or alumni community; it is not clear how well the approach we described here would work when focused on a specific citizenry.

Some institutions (or some individuals within an institution) may not consider the public to be qualified to provide input on issues they consider to be primarily technical or to require expert knowledge to assess both harms and benefits. Indeed, professionals may operate under the implicit acknowledgement that society does not understand these practices sufficiently to regulate them, and thus trusts the professionals to self-regulate in ways that follow societal values [44]. We encourage future work to address the views of both experts and non-experts (and those within and outside institutions using animals), to better understand the specific areas where public input is most desired and areas of conflict over who should have a voice.

The current study probed participant attitude using only a single question: “Do you support the use of these (insert species) for this research”. Using a series of related questions (and then calculating an attitude construct) may have improved model fit [45]. We chose not to take this approach as we were concerned about participant fatigue, but future work should consider this option. A strength of our approach was to include an open-ended question in which we asked participants to explain their responses. We suggest that our qualitative analysis of these responses helped to provide a comprehensive accounting of participants’ perspectives.

The different research scenarios we assessed varied in a number of dimensions, including species, procedure and purpose, all of which likely affected participant attitude [2–4]. The proposals were selected to provide a wide range of potential issues and thus encourage variation in participant responses. Other study designs would be required to make strong inferences regarding the importance of specific factors, for example testing scenarios that describe similar procedures applied to different animal species.

The sample we recruited was somewhat younger than the US census average [46], but as outlined above our findings on age were consistent with published literature. Our sample was also slightly female biased relative to the census. Given the interaction between research protocol and gender discussed above we encourage readers to consider the differences between research protocols separately by gender.

## Conclusions

Our online survey allowed input from a broad sample of the public with a diversity of views on the acceptability of the proposed research. Many participants provided clear input that could offer institutions a better understanding of the concerns people have regarding specific projects, helping reduce the risk that research practices are out of step with community values.

## Supporting information

S1 File Survey. (PDF)

S2 File Codebook for qualitative data analysis of participant responses to proposed animal research protocols. (XLSX)

S3 File Raw participant data. Participants (*n* = 1521) answered demographic questions and responded on a 7-point scale to the question “Do you support this use of [animals] in this research?” with animal varying (i.e. mice, pigs, monkeys or zebrafish) depending upon proposed research and text justification for answer.
(CSV)

S4 File SAS code. (TXT)
