# Supplementary material for: Public consultation in the evaluation of animal research protocols
Source: PLoS One. 2021 Dec 1;16(12):e0260114. doi: 10.1371/journal.pone.0260114 (PMC8635329; doi:10.1371/journal.pone.0260114)
Supplement: S1 File — (PDF) [file pone.0260114.s001.pdf]

Public engagement in the evaluation of animal research protocols

---

Start of Block: Consent Form

Q1.1 **Consent**

Study Title: Public engagement in the evaluation of animal research protocols

Principal Investigator: Dr. Dan Weary, Professor and NSERC Industrial Research Chair, Faculty of Land and Food Systems, University of British Columbia, dan.weary@ubc.ca 604-822-3954

Co-Investigator: Dr. Marina von Keyserlingk, Professor and NSERC Industrial Research Chair, Faculty of Land and Food Systems, University of British Columbia, marina.vonkeyserlingk@ubc.ca 604-822-4898

Co-Investigator: Michael Brunt, Graduate student, University of British Columbia, michael.brunt@ubc.ca 519-803-6587

Recruitment
Participation in this project is open to anyone 18 years of age or older. You were recruited to participate in this study using CloudResearch powered by TurkPrime. CloudResearch will not provide our research team with any of your identifying information. This survey is anonymous.

Study Procedures
You will be asked to read descriptions of one animal research protocols and then answer if you support (or not) the use of animals for this purpose. You will also be asked to provide an open-ended text response to explain your choice. This survey should take about 10 minutes or less to complete.

This survey is being conducted as part of a graduate degree. This survey collects only answers

to questions, not any personally identifying information. Data from this survey will be publicly available at the time of publishing, and as a result may be used in future research on the topic of public engagement in the evaluation of animal research protocols.

Risks
There are no known risks in taking this survey. You may leave at any point during the survey. Data from your survey will be collected and made publicly available at the time of publishing. After you submit your survey you will not be able to withdraw your data. However, only answers to questions will be collected as data and made available. No data on your personal information or identity will be collected (i.e. all data collected will be de-identified).

Confidentiality
We will not collect any information that allows us to identify or contact you individually. The data you provide will be stored in a secure database for a minimum of 5 years and will be both password protected and encrypted. Data will only be accessible to the research team until the time of publication when raw anonymous data will be made publicly available. This online survey is hosted by The Survey Tool, a cloud-based service provisioned by Qualtrics, a service provider contracted by UBC. If you choose to participate in the survey you understand that your responses will be stored in Toronto, Ontario and backed up in Montreal, Quebec. More information about the privacy and security of The Survey Tool can be found at: https://it.ubc.ca/services/teaching-learning-tools/survey-tool/qualtrics-faqs#privacy. The privacy and security policy of the survey service provider Qualtrics can be found at: http://www.qualtrics.com/privacy-statement/.

If you have any concerns or complaints about your rights as a research participant and/or your experiences while participating in this study, contact the Research Participant Complaint Line in the UBC Office of Research Ethics at 604-822-8598 or if long distance e-mail RSIL@ors.ubc.ca or call toll free 1-877-822-8598.

Consent
I am at least 18 years of age and I understand my participation in this study is entirely voluntary and that I may choose to quit at anytime. I understand that any information I provide will be anonymous and will only be stored and analyzed for the purposes of this research.

Ethics ID#: H20-01863

○ YES, I consent to participate in this study (1)

End of Block: Consent Form

Start of Block: IACUC statement

Q2.1 Whenever animals are used for university-based research, a committee evaluates the proposal and decides whether the research will be allowed. This committee includes at least one member of the public who is not associated with the university. Details of the animal-based research are not made available for public input beyond committee members. In this survey we are seeking broader public input on whether or not certain types of research should be allowed. We are asking for your input on this specific proposal.

End of Block: IACUC statement

Start of Block: Chronic pain mice

Q3.1 **Chronic pain research using mice:**

Nerve injury can lead to chronic pain characterized by a combination of symptoms including sensations of spontaneous pain, numbness, burning, as well as increased sensitivity to previously non-painful stimuli. This type of pain is known as 'neuropathic' pain and these symptoms can persist throughout life. Drugs currently available for treating neuropathic pain are of limited effectiveness and may have unwanted side effects, so research on new drugs continues. Researchers cause neuropathic pain in animals by injuring a nerve, and measuring responses to mechanical pressure, heat or cold, and chemicals.

The goal of the proposed study is to investigate the effect of a drug that inhibits calcium and calmodulin-dependent protein kinase II signalling (an enzyme that may be involved in transmission of pain signals) potentially providing humans more effective pain relief.

Researchers will perform surgery on mice to tie off two spinal nerves (connecting to the sciatic nerve), generating neuropathic pain. Presence of this pain will be evaluated by applying light pressure or heat to the affected foot (to test for increased sensitivity). These tests end as soon as the animal responds and before tissue damage can occur. These tests will be performed both on animals that have received the drug, and on control animals that have received no drug, to determine whether the drug reduces pain responses. Approximately 150 mice will be required for this study.

For more information here is an example of a similar published study. Chen F., Luo F., Yang C., Kirkmire C.M., and Wang Z.J. (2009) Acute inhibition of Ca2+/calmodulin-dependent protein kinase II reverses experimental neuropathic pain in mice. The Journal of Pharmacology and Experimental Therapeutics 330(2):650-659.

Q3.2 **Please answer the following questions:**

Q3.3 Do you support the use of these mice for this research?

- ○ No (1)
- ○ 18 (2)
- ○ 19 (3)
- ○ Neutral (4)
- ○ 21 (5)
- ○ 22 (6)
- ○ Yes (7)

Q3.4 Please explain to the University why you would or would not be willing to support this research:

____

____

____

____

____

End of Block: Chronic pain mice

Start of Block: Transplantation pig

Q4.1 **Transplantation research using pigs:**

Transplantation has become a standard treatment for the failure of hearts, lungs, kidneys and other organs in humans. One-year survival rates for heart and lung transplant recipients are good (85% and 77% respectively). Longer-term survival remains a problem and depends on the use of drugs to prevent the immune system from rejecting the transplanted organ, leaving the patient prone to serious infections. The major challenge is to find ways of stopping the immune system from destroying the donated organ. The goal is that the immune system would not recognize the donated organ as foreign (known as specific immune tolerance), but would otherwise function normally. The proposed research will use pigs to study the immunological basis and mechanisms for organ rejection; pigs share many physiological similarities with humans and are a favoured species for pre-clinical experimentation. Pigs will receive transplanted organs from other pigs and over time various tissue samples will be collected. The donor pigs are euthanized at the time of organ harvesting, and the recipient pigs will be euthanized following sample collection and monitoring for organ rejection.
For more information here is an example of a similar published study. Joffre O, Gorsse N, Romagnoli P, Hudrisier D, and van Meerwijk JP. 2004. Induction of antigen-specific tolerance to bone marrow allografts with CD4+CD25+ T lymphocytes. Blood. 103(11):4216-4221.

Q4.2 **Please answer the following questions:**

Q4.3 Do you support the use of these pigs for this research?

- No (1)
- 19 (2)
- 20 (3)
- Neutral (4)
- 22 (5)
- 23 (6)
- Yes (7)

Q4.4 Please explain to the University why you would or would not be willing to support this research:

____

____

____

____

____

End of Block: Transplantation pig

Start of Block: Smoking mice

Q5.1 **Smoking research using mice:**

Smoking during pregnancy not only causes direct adverse effects on the foetus and the newborn baby, but it has also been linked to complications later in the child's life, such as aggression, depression, anti-social behaviour, cognitive and auditory deficits and increased rates of substance abuse. The proposed research aims to use mice to understand how nicotine interferes with brain development and what effect pre/postnatal nicotine exposure has on the adolescent and adult brain. This information could be used to later devise more effective treatments of these disorders. For this research, pregnant and nursing mice will be given nicotine in their drinking water. Once the offspring are weaned, the parents will be euthanized. Their offspring will be given several behavioural tests, such as open field, object recognition, and passive-avoidance tests. The offspring will then be euthanized and the cellular architecture of their brain tissue will be examined.
For more information here is an example of a similar published study. Coddou C, Bravo E, and Eugenín J. 2009. Alterations in cholinergic sensitivity of respiratory neurons induced by pre-natal nicotine: a mechanism for respiratory dysfunction in neonatal mice. Philos Trans R Soc Lond B Biol Sci. 364(1529):2527-2535.

Q5.2 **Please answer the following questions:**

X→

Q5.3 Do you support the use of these mice for this research?

- ◯ No (1)
- ◯ 9 (2)
- ◯ 10 (3)
- ◯ Neutral (4)
- ◯ 12 (5)
- ◯ 13 (6)
- ◯ Yes (7)

Q5.4 Please explain to the University why you would or would not be willing to support this research:

____

____

____

____

____

End of Block: Smoking mice

Start of Block: Parkinson's monkeys

Q6.1 **Parkinson's research using monkeys:**

Parkinson’s disease is a neurodegenerative disorder that impairs motor skills, cognitive processes, and other functions. Individuals suffering from Parkinson’s disease are treated with drugs that help alleviate some of the symptoms, but these drugs also cause debilitating side effects. To date, no drugs exist to prevent Parkinson’s or to stop it in its course. The proposed research aims to create rhesus macaque models of Parkinson’s disease – if the model is successful, more animals will be used to study the effectiveness of novel drug therapies for

Parkinson’s disease. For this research, rhesus macaques will be injected with a protein that has been shown to cause Parkinsonian symptoms in rats. Monkeys will be housed in pairs in large cages (allowing climbing and jumping) and observed for the presence of small impairments indicative of Parkinson’s disease. They will also be asked to perform behavioural tasks to test for dexterity, movement, and reaction times. Finally, they will undergo several PET (3-D radiological image used to evaluate body function) and MRI (radiological image used to visualize detailed internal structure of the body) scans, and on occasion, samples of their cerebrospinal fluid (fluid that surrounds the brain and spinal cord) will be collected under deep anaesthesia. The monkeys will eventually be euthanized, and their brain tissue collected and analyzed.

For more information here is an example of a similar published study. Pasquereau B, and Turner RS. 2011. Primary motor cortex of the parkinsonian monkey: differential effects on the spontaneous activity of pyramidal tract-type neurons. Cerebral Cortex 21(6):1362-1378.

Q6.2 **Please answer the following questions:**

Q6.3 Do you support the use of these monkeys for this research?

- ○ No (1)
- ○ 9 (2)
- ○ 10 (3)
- ○ Neutral (4)
- ○ 12 (5)
- ○ 13 (6)
- ○ Yes (7)

Q6.4 Please explain to the University why you would or would not be willing to support this research:

____

____

____

____

____

End of Block: Parkinson's monkeys

Start of Block: Skin cancer zebrafish

Q7.1 **Skin cancer research using zebrafish:**

Animals are often used in the development and testing of drugs for the treatment of cancers, including skin cancer. One method of creating animal models for skin cancer research is to induce mutations using a process called N-ethyl-N-nitrosourea (ENU) mutagenesis. Here we present proposed research which aims to create zebrafish models of skin cancer using this method. The researchers will immerse male zebrafish in a chemical bath of ENU, causing random mutations in the germline cells (cells whose genetic material can be passed on to offspring). The zebrafish will then mate with normal females, passing on the mutation to offspring that will now be predisposed to develop tumors. These offspring will be used to test various drug treatments.
For more information here is an example of a similar published study. Stoletov K, and Klemke R. 2008. Catch of the day: zebrafish as a human cancer model. Oncogene. 27(33):4509-4520.

Q7.2 **Please answer the following questions:**

X→

Q7.3 Do you support the use of these zebrafish for this research?

- No (1)
- 9 (2)
- 10 (3)
- Neutral (4)
- 12 (5)
- 13 (6)
- Yes (7)

Q7.4 Please explain to the University why you would or would not be willing to support this research:

____

____

____

____

____

End of Block: Skin cancer zebrafish

Start of Block: General

Q8.1
For the next 7 questions:

In a similar format to the previous question, a university near you has decided to adopt a survey to get broad public input about its planned animal-based research proposals

---

Q8.2 How important is it to ask for input from the broader public?

- ○ Not important (1)
- ○ 2 (2)
- ○ 3 (3)
- ○ Neutral (4)
- ○ 5 (5)
- ○ 6 (6)
- ○ Important (7)

Q8.3 Why?

____

____

____

____

____

Q8.4 In your view, how likely is it that the broader public would take the time to provide useful input?

- Unlikely (1)
- 2 (2)
- 3 (3)
- Neutral (4)
- 5 (5)
- 6 (6)
- Likely (7)

Q8.5 Please explain what you would consider to be useful input, from the broader public, on planned animal-based research proposals

____

____

____

____

____

Q8.6 How likely is the university to benefit by requesting input from the broader public?

- Unlikely (1)
- 2 (2)
- 3 (3)
- Neutral (4)
- 5 (5)
- 6 (6)
- Likely (7)

Q8.7 How likely is the broader public to benefit by being involved?

- Unlikely (1)
- 2 (2)
- 3 (3)
- Neutral (4)
- 5 (5)
- 6 (6)
- Likely (7)

Q8.8 How likely are the animals to be used in the research to benefit by requesting input from the broader public?

- ◯ Unlikely (1)
- ◯ 2 (2)
- ◯ 3 (3)
- ◯ Neutral (4)
- ◯ 5 (5)
- ◯ 6 (6)
- ◯ Likely (7)

End of Block: General

Start of Block: Pandemic other

X→

Q9.1 How do you think the COVID-19 pandemic has influenced the average American's willingness to support animal-based research?

- ◯ Less supportive (1)
- ◯ 12 (2)
- ◯ 13 (3)
- ◯ Neutral (4)
- ◯ 15 (5)
- ◯ 16 (6)
- ◯ More Supportive (7)

End of Block: Pandemic other

Start of Block: Pandemic self

Q10.1 How do you think the COVID-19 pandemic has influenced your willingness to support animal-based research?

- Less supportive (1)
- 13 (2)
- 14 (3)
- Neutral (4)
- 16 (5)
- 17 (6)
- More supportive (7)

End of Block: Pandemic self

Start of Block: IMC

Q11.1 **Animals** Most modern theories of psychology recognize the fact that perceptions do not take place in a vacuum. Individual preferences and knowledge, along with situational variables can greatly impact the perception process. In order to facilitate our research on perceptions we are interested in knowing certain factors about you, the perciever. Specifically, we are interested in whether you actually take the time to read the directions; if not, then some of our questions that rely on changes in the instructions will be ineffective. So, in order to demonstrate that you have read the instructions, please ignore the animals below. Instead, simply check the "Other" option and in the corresponding box, enter the text: I read the instructions

Which of these animals are used for research?

(check all that apply)

- [ ] Pig (1)
- [ ] Cow (2)
- [ ] Horse (3)
- [ ] Cat (4)
- [ ] Sheep (5)
- [ ] Dog (6)
- [ ] Other: (7) ____

End of Block: IMC

Start of Block: Demographic Questions

Q12.1 **In this last section of the survey, please answer the following demographic questions:**

---

*

Q12.2 Age:

____

X→

Q12.3 Gender:

- Woman (1)
- Man (2)
- Transgender woman (3)
- Transgender man (4)
- Gender not listed (5)
- Prefer not to say (6)

Q12.4 Do you have any children?

- Yes (1)
- No (2)

Q12.5 Do you have any pets?

- Yes (1)
- No (2)

Q12.6 What is the highest level of education that you have?

- Less than high school diploma (1)
- High school graduate (high school diploma or equivalent including GED) (2)
- Some college, but no degree (3)
- Associate degree in college (2-year) (4)
- Bachelor's degree in college (4-year) (5)
- Post-graduate or professional degree (e.g. Master's, PhD, MD, DVM) (6)

Q12.7 Please indicate the answer that includes your entire household income (previous year) before taxes:

- Less than $25,000 (1)
- $25,000-$34,999 (2)
- $35,000-$49,999 (3)
- $50,000-$74,999 (4)
- $75,000-$99,999 (5)
- $100,000-$149,999 (6)
- $150,000 or more (7)

Q12.8 Which of the following best describes the area where you have lived most of your life?

- Urban (1)
- Suburban (2)
- Rural (3)

Q12.9 Which statement best describes your diet?

- I do not eat meat but do eat other animal products such as milk products and eggs (vegetarian diet) (1)
- I do not eat animal products (vegan diet) (2)
- I eat meat and other animal products (omnivore diet) (3)

Q12.10 Which best describes your political views?

- Conservative (1)
- Moderate (2)
- Liberal (3)
- None of the above (4)
- No opinion (5)

Q12.11 How familiar are you with animal-based research?

- Not at all familiar (1)
- Not very familiar (2)
- Slightly familiar (3)
- Somewhat familiar (4)
- Very familiar (5)
- Extremely familiar (6)

Q12.12 Are you involved involved in animal-based research?

- Yes (1)
- No (2)

Q12.13 Please feel free to leave any feedback below on the topics of this survey or on the survey itself:

____

____

____

____

____

End of Block: Demographic Questions
